# The COVID-19 pandemic in the eyes of Polish and Slovak medical students

**DOI:** 10.3389/fpubh.2026.1821945

**Published:** 2026-05-28

**Authors:** Józefa Dąbek, Radka Kurucová, Katarína Žiaková, Magdalena Szynal, Martina Tomagová

**Affiliations:** 1 Department of Cardiology, Faculty of Health Sciences in Katowice, Medical University of Silesia in Katowice, Katowice, Poland; 2 Department of Nursing, Jessenius Faculty of Medicine in Martin, Comenius University in Bratislava, Martin, Slovakia

**Keywords:** COVID-19, medical students, pandemic, Poland, Slovakia

## Abstract

**Background:**

The main aim of the study was to assess the perception of the COVID-19 pandemic by Polish and Slovak medical students. Additional aims were assessment of their satisfaction with the quality of health care services and their attitudes toward vaccination against SARS-CoV-2. A group of 635 (100%) students of nursing and medicine from Poland and Slovakia were surveyed.

**Methods:**

The results are part of a study conducted as part of international cooperation between the Silesian Medical University in Katowice (Poland) and the Comenius University in Bratislava, Jessenius Faculty of Medicine in Martin (Slovakia). Data were collected using a proprietary questionnaire developed for the purposes of this study.

**Conclusion:**

The findings provide exploratory insight into retrospective perceptions related to psychosocial experiences during the COVID-19 pandemic among surveyed medical students from Poland and Slovakia. The lower evaluation of healthcare services during the pandemic and differences observed between Poland and Slovakia may reflect the perceived strain on healthcare systems, although these results should be interpreted with caution. While students expressed confidence in vaccination, differences between declared attitudes and vaccination uptake were observed, which may highlight the possible role of targeted educational and communication strategies. Overall, the findings may suggest the importance of considering psychosocial and systemic factors in shaping health-related attitudes among medical students. However, due to study limitations, the results should be interpreted cautiously and are not fully generalizable and should primarily be understood within the context of the analyzed sample.

## 1 Introduction

The COVID-19 pandemic, declared by the World Health Organization (WHO) in March 2020, has had a significant and long-lasting impact on the functioning of healthcare systems, population health behaviors, and the education of medical personnel (1). Among medical students, who will form the backbone of the future healthcare system, attitudes toward vaccinations and the assessment of healthcare quality are crucial. Understanding these aspects is crucial not only for public health but also for planning effective educational and preventive strategies in the academic community (2–4).

In Poland, the first case of COVID-19 was confirmed on March 4, 2020. In the following months, a nationwide lockdown, mandatory face coverings, and mobility restrictions were introduced. A mass vaccination program was launched in 2021, initially targeting healthcare workers and medical students (5, 6). By mid-2023, over 6.5 million infections and approximately 120,000 deaths had been recorded, significantly burdening the healthcare system (7). Poland was among the countries with moderate vaccination coverage, and by the end of 2022, approximately 59% of the population had completed the full vaccination cycle (8).

In Slovakia, the first case of SARS-CoV-2 infection was recorded on March 6, 2020. The government immediately introduced a state of emergency from March 16 to June 13, 2020, which included mobility restrictions and restrictions on higher education. In 2020-2021, the country implemented a unique mass testing strategy in Europe, including a screening campaign of several million people (9). Despite these measures, Slovakia experienced several strong waves of disease, and the coronavirus death rate was among the highest in the European Union (10). In 2022, the full vaccination rate was approximately 49%, one of the lowest in the region (11).

Previous studies have shown that medical students had a relatively high level of knowledge about COVID-19, which translated into their more frequent use of preventive measures, such as hand hygiene, social distancing, and wearing masks (12–14). In accordance with the assumptions of Health the Belief Model (HBM), the perceived risk of disease and the belief in the effectiveness of protective measures played an important role in preventing the spread of infections and the development of the disease (15).

Attitudes toward vaccinations proved to be a particular challenge. Despite the hypothetical high level of medical knowledge among medical students, significant differences in acceptance of COVID-19 vaccinations were observed. According to global reviews, acceptance rates among medical students ranged from 50% to over 85%. In Poland, studies conducted among students and healthcare professionals revealed relatively high vaccination rates, but also hesitancy toward booster doses, most often resulting from concerns about side effects or beliefs about the low risk of severe disease (6, 16). In Slovakia, lower vaccination acceptance rates were observed, which was attributed, for example, to limited public trust in state institutions and the influence of disinformation (9, 11).

The pandemic has changed the way healthcare services are provided and perceived. Poland and Slovakia have seen rapid development of telemedicine, which has allowed for the maintenance of continuity of care while limiting in-person contact (17). Various studies have shown that patient satisfaction with telemedicine services was relatively high, but some respondents indicated a deterioration in access to traditional services and a decline in trust in healthcare institutions (7, 8, 18).

In our study the perception of the COVID-19 pandemic was operationalized through three complementary dimensions: evaluation of healthcare services, attitudes toward vaccination, and subjective experiences related to the pandemic (both positive and negative). This approach reflects the multidimensional nature of health-related perceptions, encompassing cognitive (evaluation of healthcare), behavioral (vaccination attitudes), and psychosocial (pandemic experiences) components. Such an understanding is consistent with health psychology frameworks, including the Health Belief Model, which emphasizes the role of perceived risk, beliefs, and contextual factors in shaping health-related attitudes and behaviors.

### 1.1 The aim of the study

The main aim of the study was to assess the perception of the COVID-19 pandemic by Polish and Slovak medical students.

Additional aims were:

1. Assessment of the satisfaction of surveyed students with the quality of health care services.
2. Assessment of the attitudes of surveyed students toward vaccination against SARS-CoV-2.

## 2 Materials and methods

This study was designed as an exploratory, retrospective, cross-sectional analysis. The primary aim was to describe and compare perceptions among groups rather than to establish causal relationships or develop predictive models.

The research was initiated after obtaining the opinions of the Bioethics Committees of the Silesian Medical University in Katowice (no. BNW/NWN/0052/KB/252/24) and the Comenius University in Bratislava (no. EK 122/2024). The results presented in this paper are part of a study conducted as part of international cooperation between the Silesian Medical University in Katowice (Poland) and the Comenius University in Bratislava, Jessenius Faculty of Medicine in Martin (Slovakia), regarding the impact of the COVID-19 pandemic on Polish and Slovak medical students.

A group of 635 (100%) students of nursing and medicine from Poland and Slovakia were surveyed. The selection of students from Poland and Slovakia was motivated by several considerations. While both countries are geographically and culturally similar, they differ in the organization of their healthcare systems, public communication strategies, and the course of actions related to the COVID-19 pandemic, including vaccination campaigns. We believed that this comparison offered the opportunity to capture subtle differences in attitudes and perceptions of the pandemic while limiting the impact of significant cultural differences. In southern Poland (Silesia) where the study was conducted, and in the study region of Slovakia, there are an estimated 13,000–16,000 students studying medicine and nursing. To be representative, the sample should be approximately 375 students from this population. Our study includes 635. However, participants were recruited using a non-probability convenience sampling method based on voluntary participation (self-selection). While this approach facilitated efficient data collection, it does not ensure clearly representativeness of the sample and may be associated with selection bias. Therefore, the findings should be interpreted with caution and cannot be fully generalized to the broader population.

The data used in this study were collected in 2024-2025, after the COVID-19 pandemic had ended. The study included students who were enrolled at universities at the time of the survey but did not study during the most critical phases of the COVID-19 pandemic. Therefore, the results reflect a retrospective assessment of pandemic experiences and current attitudes toward vaccination and the functioning of the healthcare system in the post-pandemic period.

Data were collected using a proprietary (author-developed) tool designed specifically for the purposes of this study. The tool was developed based on a review of the literature and the study objectives. The research tool contained questions regarding demographic data, the quality of medical services in Poland and Slovakia, vaccinations against SARS-CoV-2, and the negatives and positives of the pandemic. Questions regarding the quality of medical services included scores from 1 (worst) to 5 (best), which respondents assigned to evaluate a given service during and outside the pandemic, with the result being the average score.

Its content was reviewed by the research team to ensure relevance and clarity, and a pilot test was conducted among a small group of students to verify comprehensibility. The pilot study was conducted to verify whether the questions were understandable and consistent with the intentions of the researchers. However, the questionnaire was not subjected to full psychometric validation, which should be considered when interpreting the results. We would not be able to calculate any coefficient from our tool because not all questions have a graduated scale.

The questionnaires were anonymous and voluntary. All students included in the study consented to completing the questionnaire. The surveys were administered in paper form during classes and lectures in the nursing and medical programs at the Silesian Medical University in Katowice (Poland) and Comenius University in Bratislava, Jessenius Faculty of Medicine in Martin (Slovakia). The surveys were collected in sealed boxes, and the data were then entered into an Excel spreadsheet.

Statistical analyses were performed using Statistica software. Ver. 13.3 (Statsoft, Poland). Qualitative data were presented using counts and percentages, while quantitative data were presented using descriptive statistics. Significance analyses of differences between groups were performed using the following tests: Chi-square (significance in the number of respondents), Mann- Whitney *U*, and Kruskall-Wallis (significance in quantitative data). The level of statistical significance was set at *p* < 0.05.

## 3 Results

The general characteristics of the study group are presented in Table 1.

**Table 1:** General characteristics of the study group.

The entire study group (635; 100%)							
Variables						*n*	%
Origin					Poland	431	67.87
					Slovakia	204	32.13
Sex					Women	528	83.15
					Men	107	16.85
Age (years)					18-19	193	30.39
*M*	SD	Me	Q1	Q3	20-21	286	45.04
21	2	20	19	21	>21	156	24.57
Education					Secondary	543	85.51
					Higher	92	14.49
Occupation performed/apprenticeship					Nursing	379	59.69
					Medicine	256	40.31
Domicile					City	407	64.20
					Village	228	35.80
Marital status					Single/bachelor	625	98.43
					Married	7	1.10
					Divorced woman/divorced man	3	0.47

*M*, mean; SD, standard deviation; Me, median; Q1, lower quartile; Q3, upper quartile, *n*, number.

The vast majority of the study group were women (528; 83.15%). Two-thirds were Poles (431; 67.87%). The average age of the respondents was 20 years (SD = 2). Most respondents (543; 85.51%) declared secondary education, and two-thirds (407, 64.20%) lived in cities. Almost all were single (single/bachelor—625; 98.43%).

The opinions of the surveyed students regarding the quality of medical services provided in Poland and Slovakia during the pandemic and beyond are presented in Table 2.

**Table 2:** Opinions of surveyed students regarding the quality of medical services provided in Poland and Slovakia during the pandemic and beyond.

Study group (635; 100%)				
Variables	Time relative to the pandemic	Poland	Slovakia	*p* ^2^
Availability of a specialist doctor	While the pandemic	2.68 ± 1.03 (95% CI: 0.97–1.11)	3.02 ± 1.07 (95% CI: 0.97–1.19)	<0.001 (*d* = 0.27)
	Outside the pandemic	3.27 ± 0.98 (95% CI: 0.91–1.05)	2.64 ± 0.99 (95% CI: 0.90–1.10)	<0.001 (*d* = 0.15)
	*p* ^1^	<0.001 (*d* = 0.5)	<0.001 (*d* = 0.37)	—
Waiting time in the Emergency Room	While the pandemic	2.50 ± 0.99 (95% CI: 0.93–1.06)	3.15 ± 1.06 (95% CI: 0.97–1.18)	<0.001 (*d* = 0.16)
	Outside the pandemic	2.78 ± 0.93 (95% CI: 0.87–1.00)	3.10 ± 1.13 (95% CI: 1.03–1.25)	<0.001 (*d* = 0.29)
	*p* ^1^	<0.001 (*d* = 0.34)	0.478 (*d* = 0.05)	—
Availability of registration by phone	While the pandemic	3.06 ± 1.06 (95% CI: 1.00–1.14)	2.36 ± 1.06 (95% CI: 0.97–1.18)	<0.001 (*d* = 0.31)
	Outside the pandemic	3.16 ± 0.99 (95% CI: 0.93–1.06)	2.44 ± 1.01 (95% CI: 0.92–1.12)	<0.001 (*d* = 0.29)
	*p* ^1^	<0.01 (*d* = 0.13)	0.270 (*d* = 0.52)	—
Kindness of nurses	While the pandemic	3.27 ± 0.94 (95% CI: 0.88–1.01)	2.78 ± 0.93 (95% CI: 0.85–1.03)	<0.001 (*d* = 0.32)
	Outside the pandemic	3.37 ± 0.90 (95% CI: 0.84–0.96)	2.68 ± 0.99 (95% CI: 0.91–1.10)	<0.001 (*d* = 0.23)
	*p* ^1^	<0.001 (*d* = 0.13)	0.06 (*d* = 0.53)	—
Professionalism and efficiency in performing tests and procedures by nurses	While the pandemic	3.54 ± 0.92 (95% CI: 0.86–0.99)	2.59 ± 0.89 (95% CI: 0.81–0.98)	<0.001 (*d* = 0.48)
	Outside the pandemic	3.53 ± 0.90 (95% CI: 0.84–0.96)	2.44 ± 0.86 (95% CI: 0.79–0.96)	<0.001 (*d* = 0.43)
	*p* ^1^	0.839 (*d* = 0.01)	<0.001 (*d* = 0.24)	—
Respect for Patient Rights	While the pandemic	3.48 ± 0.95 (95% CI: 0.89–1.01)	2.64 ± 0.96 (95% CI: 0.88–1.07)	<0.001 (*d* = 0.47)
	Outside the pandemic	3.58 ± 0.91 (95% CI: 0.85–0.97)	2.56 ± 1.21 (95% CI: 1.10–1.34)	<0.001 (*d* = 0.36)
	*p* ^1^	<0.001 (*d* = 0.15)	<0.01 (*d* = 0.21)	—
The kindness of doctors	While the pandemic	3.30 ± 0.92 (95% CI: 0.86–0.99)	2.89 ± 0.99 (95% CI: 0.90–1.10)	<0.001 (*d* = 0.35)
	Outside the pandemic	3.40 ± 0.91 (95% CI: 0.85–0.97)	2.64 ± 0.92 (95% CI: 0.84–1.02)	<0.001 (*d* = 0.18)
	*p* ^1^	<0.001 (*d* = 0.17)	<0.001 (*d* = 0.26)	—
The level and professionalism of medical assistance	While the pandemic	3.43 ± 0.91 (95% CI: 0.85–97)	2.45 ± 0.96 (95% CI: 0.87–1.06)	<0.001 (*d* = 0.55)
	Outside the pandemic	3.50 ± 0.88 (95% CI: 0.82–0.94)	2.26 ± 0.87 (95% CI: 0.80–0.97)	<0.001 (*d* = 0.43)
	*p* ^1^	<0.01 (*d* = 0.14)	0.001 (*d* = 0.23)	—
Availability of the attending physician	While the pandemic	3.06 ± 0.94 (95% CI: 0.88–1.01)	2.93 ± 0.99 (95% CI: 0.90–1.09)	0.138 (*d* = 0.37)
	Outside the pandemic	3.27 ± 0.92 (95% CI: 0.86–0.98)	2.48 ± 0.87 (95% CI: 0.79–0.96)	<0.001 (*d* = 0.06)
	*p* ^1^	<0.001 (*d* = 0.28)	<0.001 (*d* = 0.43)	—
Taking care to maintain the patient’s privacy during the procedure	While the pandemic	3.47 ± 0.90 (95% CI: 0.84–0.96)	2.88 ± 1.09 (95% CI: 0.99–1.21)	<0.001 (*d* = 0.42)
	Outside the pandemic	3.59 ± 0.87 (95% CI: 0.81–0.93)	2.65 ± 0.93 (95% CI: 0.84–1.03)	<0.001 (*d* = 0.24)
	*p* ^1^	<0.001 (*d* = 0.2)	<0.001 (*d* = 0.25)	—
Waiting time for a doctor’s appointment in front of the office	While the pandemic	2.85 ± 0.99 (95% CI: 0.92–1.06)	3.03 ± 1.01 (95% CI: 0.92–1.12)	0.01 (*d* = 0.09)
	Outside the pandemic	2.99 ± 0.95 (95% CI: 0.89–1.02)	2.77 ± 1.08 (95% CI: 0.98–1.19)	0.02 (*d* = 0.10)
	*p* ^1^	<0.001 (*d* = 0.2)	<0.001 (*d* = 0.26)	—
Staff collaboration with family/caregiver (providing information, support, education)	While the pandemic	3.33 ± 0.91 (95% CI: 0.85–0.97)	2.68 ± 0.91 (95% CI: 0.83–1.01)	<0.001 (*d* = 0.43)
	Outside the pandemic	3.40 ± 0.88 (95% CI: 0.82–0.94)	2.50 ± 0.81 (95% CI: 0.74–0.89)	<0.001 (*d* = 0.31)
	*p* ^1^	0.01 (*d* = 0.12)	0.001 (*d* = 0.23)	—

*p*^1^, Statistical significance of the Wilcoxon signed-rank test; *p*^2^, statistical significance level of the Kruskall-Wallis test of the significance of differences between multiple independent values; *d*, Cohen’s *d* coefficient; *p*, statistical significance.

The analysis of medical students’ assessments of the provision of medical services in Poland and Slovakia showed statistically significant differences between the countries, both during the COVID-19 pandemic and beyond (*p* < 0.05).

During the pandemic, students rated the quality of services in Poland higher than in Slovakia in most analyzed categories. This applies to, among others, nurses’ courtesy (Poland: 3.27 ± 0.94 vs. Slovakia: 2.78 ± 0.93; *p* < 0.001), nurses’ professionalism in performing examinations and procedures (3.54 ± 0.92 vs. 2.59 ± 0.89; *p* < 0.001), respect for patients’ rights (3.48 ± 0.95 vs. 2.64 ± 0.96; *p* < 0.001), doctors’ courtesy (3.30 ± 0.92 vs. 2.89 ± 0.99; *p* < 0.001), and medical assistance’s professionalism (3.43 ± 0.91 vs. 2.45 ± 0.96; *p* < 0.001). These results indicate that Polish respondents assessed professional competences and interpersonal relations in medical facilities more positively. However, most observed differences were associated with small-to-moderate or moderate effect sizes, suggesting that some statistically significant differences may also be relevant from a practical perspective.

In Slovakia, during the pandemic, higher scores were given to organizational aspects, such as the availability of a specialist doctor (3.02 ± 1.07 vs. Poland 2.68 ± 1.03; *p* < 0.001) and waiting time in the emergency room (3.15 ± 1.06 vs. 2.50 ± 0.99; *p* < 0.001), which may reflect perceived better availability of services and shorter waiting times in the Slovak system during the pandemic. However, some statistically significant findings were characterized by relatively small effect sizes, indicating limited practical significance despite statistical significance.

Outside the pandemic, both countries reported higher ratings for service provision, but Poland maintained its lead in most of the surveyed categories. Students in Poland rated higher, among other things, nurses’ courtesy (3.37 ± 0.90 vs. Slovakia 2.68 ± 0.99; *p* < 0.001), nurses’ professionalism (3.53 ± 0.90 vs. 2.44 ± 0.86; *p* < 0.001), respect for patients’ rights (3.58 ± 0.91 vs. 2.56 ± 1.21; *p* < 0.001), doctors’ courtesy (3.40 ± 0.91 vs. 2.64 ± 0.92; *p* < 0.001), level of medical assistance (3.50 ± 0.88 vs. 2.26 ± 0.87; *p* < 0.001) and care for patients’ privacy (3.59 ± 0.87 vs. 2.65 ± 0.93; *p* < 0.001).

In Slovakia, outside the pandemic period, the highest scores were given to the availability of a specialist doctor (2.64 ± 0.99 vs. Poland 3.27 ± 0.98; *p* < 0.001) and the waiting time in front of the office (2.77 ± 1.08 vs. Poland 2.99 ± 0.95; *p* = 0.02).

Vaccination against SARS-C oV-2 by the surveyed students is presented in Table 3.

**Table 3:** Vaccination against SARS-CoV-2 by the surveyed students.

Study group (635; 100%)						
Variables	Yes	No	Chi^2^	*p* (*φ*)	Odds ratio	95% CI
Poland (431; 100%)	325 (75.41%)	106 (24.59%)	5,738	0.01 (0.1)	0.59	0.38–0.91
Slovakia (204; 100%)	171 (83.82%)	33 (16.18%)				
Sum (635; 100%)	496 (78.11%)	139 (21.89%)	—			

Chi^2^, value of the Chi^2^ statistic; *p*, statistical significance; φ, Crammer’s *V* coefficient; 95% CI, 95% confidence interval.

A higher vaccination rate against SARS-CoV-2 was observed among Slovak students (171; 83.82%) than among Polish students (325; 75.41%). There was a small significant difference between the nationality and vaccination against SARS-CoV-2 (*p* = 0.01) with the vaccinated students having 0.59 times the odds of the vaccination than the people not vaccinated (95% CI: 0.38, 0.91).

The reasons for not getting vaccinated against SARS-CoV-2 given by the surveyed students are presented in Table 4.

**Table 4:** Reasons for not vaccinating against SARS-CoV-2 given by surveyed students.

Respondents not vaccinated against SARS-CoV-2 (*n* = 139; 100%)						
Variables	Poland (106; 100%)		Slovakia (33; 10%)		Total (139; 100%)	
	*n*	%	*n*	%	*n*	%
Fear of side effects	9	8.49	13	39.39	22	16
Views	5	4.72	8	24.24	13	9
No justification was provided	82	77.36	7	21.21	89	64
Negative attitudes toward vaccinating loved ones	4	3.77	7	21.21	11	8
I am a convalescent	7	6.60	0	0.00	7	5
Disease that precludes vaccination	0	0.00	10	30.30	10	7
No proven effectiveness	4	3.77	1	3.03	5	4

*n*, Count; %, Percent.

The largest group of respondents who did not get vaccinated against SARS-CoV-2 did not provide a justification for their decision (Polish students: 82; 77.36%). Among students from Slovakia, the most common answers were: fear of side effects (13; 39.39%), or a disease that precluded vaccination (10; 30.30%).

The reasons for vaccination against SARS-CoV-2 given by the surveyed students are presented in Table 5.

**Table 5:** Reasons for vaccination against SARS-CoV-2 given by surveyed students.

Respondents vaccinated against SARS-CoV-2 (*n* = 496; 100%)						
Variables	Poland (325; 100%)		Slovakia (171; 100%)		Total (496; 100%)	
	*n*	%	*n*	%	*n*	%
Belief in the effectiveness of the vaccine	186	57.23	105	61.40	291	58.67
The desire to protect loved ones	74	22.77	45	26.32	119	23.99
The need to go abroad	95	29.23	9	5.26	104	20.97
Vaccination required for work	22	6.77	27	15.79	49	9.88
Working in healthcare	17	5.23	30	17.54	47	9.48
Doctor’s recommendation	16	4.92	2	1.17	18	3.63
Working abroad	5	1.54	8	4.68	13	2.62
Fear of the opinions of other vaccinated people	7	2.15	2	1.17	9	1.81

*n*, Count; %, Percent.

The most common reason for vaccination among Slovak and Polish students was the belief in the vaccine’s effectiveness (Slovakia: 105; 61.4%, Poland: 186; 57.23%).

The negative effects of the COVID-19 pandemic declared by the surveyed students are presented in Table 6.

**Table 6:** Negative effects of the COVID-19 pandemic declared by surveyed students.

The entire study group (635; 100%)						
Variables	Poland (431; 100%)		Slovakia (204; 100%)		Total (635; 100%)	
	*n*	%	*n*	%	*n*	%
No parties/social gatherings	282	65.43	128	62.75	410	64.57
Locked in the house	232	53.83	90	44.12	322	50.71
Quarrels in the family	144	33.41	95	46.57	239	37.64
Closing of the restaurant	153	35.49	63	30.88	216	34.02
Deterioration of mental state	144	33.41	48	23.53	192	30.24
Lack of motivation to learn	83	19.26	61	29.90	144	22.68
Closure of clubs/bars	106	24.59	24	11.76	130	20.47
Closure of sports facilities	89	20.65	35	17.16	124	19.53
Weight gain	66	15.31	46	22.55	112	17.64
Loss of job	32	7.42	23	11.27	55	8.66
Increase/start smoking	15	3.48	7	3.43	22	3.46
Increasing/starting to consume larger amounts of alcohol	8	1.86	5	2.45	13	2.05
No negative effects	1	0.23	9	4.41	10	1.57

*n*, Count; %, Percent.

The most common negative effect of the pandemic for all surveyed student subgroups was the lack of events/social gatherings (Slovakia: 128; 62.75%, Poland: 282; 65.43%).

Common responses included being confined to the house, family quarrels, restaurant closures, and a deterioration of mental health and lack of motivation to learn.

The places and activities that the surveyed students missed the most during the COVID-19 pandemic are presented in Table 7, while the most troublesome restrictions are presented in Table 8.

**Table 7:** Places and activities that the surveyed students missed the most during the COVID-19 pandemic.

The entire study group (635; 100%)						
Variables	Poland (431; 100%)		Slovakia (204; 100%)		Total (635; 100%)	
	*n*	%	*n*	%	*n*	%
Restaurants	113	26.22	38	18.63	151	23.78
Clubs/bars	67	15.54	28	13.73	95	14.96
Parties/social gatherings	297	68.91	99	48.53	396	62.36
Sports facilities	83	19.26	39	19.12	122	19.21
Full-time studies	63	14.62	71	34.80	134	21.10
Nothing was missing	42	9.74	39	19.12	81	12.76

*n*, Count; %, Percent.

**Table 8:** The most burdensome restrictions declared during the COVID-19 pandemic by the surveyed students.

The entire study group (635; 100%)						
Variables	Poland (431; 100%)		Slovakia (204; 100%)		Total (635; 100%)	
	*n*	%	*n*	%	*n*	%
Ban on parties/social gatherings	218	50.58	142	69.61	360	56.69
Remote learning	116	26.91	58	28.43	174	27.40
Closing of shops	158	36.66	70	34.31	228	35.91
Wearing masks	120	27.84	92	45.10	212	33.39
Isolation	248	57.54	57	27.94	305	48.03
Social distancing	122	28.31	2	0.98	124	19.53

*n*, Count; %, Percent.

For both Slovak and Polish students, the most troubling restriction during the pandemic proved to be the ban on parties and social gatherings. The same was true for the lack of places and activities—students in these areas also most frequently indicated that the lack of parties and social gatherings was what they felt most acutely.

The positive effects of the COVID-19 pandemic declared by the surveyed students are presented in Table 9.

**Table 9:** Positive effects of the COVID-19 pandemic declared by surveyed students.

The entire study group (635; 100%)							
Variables		Poland (431; 100%)		Slovakia (204; 100%)		Total (635; 100%)	
		*n*	%	*n*	%	*n*	%
Improving the organization of everyday life		97	22.51	80	39.22	177	27.87
Increasing the amount of time spent on	Reading books	182	42.23	85	41.67	42.05	42.05
	Hobbies	107	24.83	66	32.35	27.24	27.24
	Self-development	170	39.44	106	51.96	43.46	43.46
	Family life	167	38.75	120	58.82	45.20	45.20
	Rest	71	16.47	21	10.29	14.49	14.49
House renovation		74	17.17	93	45.59	167	26.30
No need to commute to work		162	37.59	20	9.80	182	28.66
No positive effects		69	16.01	1	0.49	70	11.02

*n*, Count; %, Percent.

Slovak students most often responded that during the pandemic they had more time to develop their interests (106; 51.96%) and family life (120; 58.82%).

Polish students, on the other hand, most often indicated that they had more time to read books (182; 42.33%).

The activities on which surveyed students spent more time during the COVID-19 pandemic are presented in Table 10.

**Table 10:** Activities on which the surveyed students devoted more time during the pandemic COVID-19.

The entire study group (635; 100%)						
Variables	Poland (431; 100%)		Slovakia (204; 100%)		Total (635; 100%)	
	*n*	%	*n*	%	*n*	%
Self-care	242	56.15	148	72.55	390	61.42
Self-development	243	56.38	118	57.84	361	56.85
Taking care of the family	138	32.01	125	61.27	263	41.42
Studying	149	34.57	87	42.65	236	37.17
Cooking	72	16.71	47	23.04	119	18.74

*n*, Count; %, Percent.

Both Slovak and Polish students spent most of their time during the COVID-19 pandemic on self-care (Slovakia: 148; 72.55%, Poland: 242; 56.15%) and self-development (Slovakia: 118; 57.84%, Poland: 243; 56.38%).

## 4 Discussion

Due to the exploratory, retrospective, and cross-sectional design of the study, the findings should be interpreted as descriptive observations rather than evidence of causal relationships.

In our study, medical students in Poland rated the quality of healthcare services significantly higher than their Slovak counterparts, both during the COVID-19 pandemic and beyond. During the pandemic, facilities in Poland received higher ratings, particularly in terms of staff professional competence, courtesy, and respect for patient rights (*p* < 0.001). In Slovakia, however, organizational aspects, such as the availability of specialists and waiting times for services, were rated more favorably (*p* < 0.001).

Outside the pandemic, ratings in both countries improved, but Poland maintained its advantage in most categories related to the quality of contact with staff and professionalism of services (*p* < 0.001). In Slovakia, the highest ratings were given to elements related to the availability of services (*p* < 0.001; *p* = 0.02).

In Poland, the pandemic period was associated with deterioration in the availability of inpatient and outpatient services (19). According to a study by Tuczyńska et al. (20), the reorganization of facility operations negatively impacted the assessment of service quality and patient satisfaction. Patients also experienced difficulties registering appointments and contacting doctors. Mularczyk-Tomczewska et al. (21), however, noted that the most important factors influencing patient satisfaction in Poland were: the quality of communication, the availability of specialists, and the competence of medical personnel.

In Slovakia, research by Gavurová et al. (22) revealed that patient satisfaction in Slovak hospitals was largely dependent on organizational and infrastructural factors, which were underfunded in many facilities. Furthermore, analyses by Jenčová et al. (23) showed that the operational efficiency of Slovak hospitals during the crisis period decreased significantly compared to the pre-pandemic period.

Particularly noteworthy is the deterioration in the assessment of the functioning of medical services during the pandemic in our own study, in a group of Polish students, in almost all aspects. Śliwczyński et al. (19) demonstrated that the COVID-19 pandemic reversed the previous trend of increasing the number of medical services, and the healthcare system operated with limited capacity, which could have reduced public confidence in its quality. The presented results are consistent with the research by Izdebski et al. (24), who noted a deterioration in communication between the patient and the healthcare system, and with the analyses by Kludacz-Alessandri et al. (25), according to which patients reported lower satisfaction with in-person visits but had relatively positive experiences with teleconsultations.

In the case of Slovakia, Pacutová et al. (26) described that crisis management in healthcare during the pandemic placed a heavy burden on healthcare workers, affecting the quality of their work and relationships with patients. In turn, Puteková et al. (27) noted that nurses in Slovakia experienced increased stress and worsening working conditions, which could translate into lower assessments of service quality by students observing the functioning of the system from the inside. Also interesting in this context are the results of the study by Halama et al. (28), who, analyzing the experiences of patients in Slovakia, emphasized the sense of distance in contacts with staff (“doctors looked like aliens”).

Our study showed a higher rate of vaccination against SARS-CoV-2 among Slovak students (171; 83.82%) than Polish students (325; 75.41%). However, the largest group of unvaccinated surveyed individuals did not provide a justification for not getting vaccinated against SARS-CoV-2 (Polish students: 82; 77.36%). Among Slovak students, the most common answers were: fear of side effects (13; 39.39%) or a disease that precludes vaccination (10; 30.30%). The most common reason for getting vaccinated was belief in the vaccine’s effectiveness (Slovakia: 105; 61.4%, Poland: 186; 57.23%).

Various European and Polish studies have observed high acceptance of vaccinations among medical personnel and medical students, particularly physicians and medical students (17, 29). Lindner-Pawłowicz et al. (29) showed that over 75% of medical students and 80% of physicians in Poland declared confidence in vaccinations against COVID-19, while among nurses this level was significantly lower (34.5%). Similar results were obtained by Dziedzic et al. (17), who found that 93.7% of healthcare workers had received at least one dose of the vaccine, and 74.5% were willing to receive a booster dose. The main motivations for vaccination included protecting one’s own health and that of loved ones, while fear of adverse effects was a discouraging factor (17, 30). Slavec et al.’s (31) research revealed that hesitancy toward vaccinations was mainly determined by: lack of confidence in the benefits of vaccination, concerns about side effects, and a preference for “natural immunity”. Among students and healthcare workers, trust in public institutions and government communication were key factors in the decision to vaccinate (32, 33). Hoffmann et al. (34), in turn, noted that vaccinated individuals more often reported lower sick leave and higher productivity, which may indicate the practical dimension of vaccination effectiveness in professional populations.

Vaccine hesitancy is currently recognized as a complex and multidimensional phenomenon influenced by psychological, social, and systemic factors (35). Recent reviews indicate that its prevalence remains substantial, affecting approximately one-third of the population globally (16). Importantly, hesitancy is also observed among medical students and healthcare professionals, despite their medical knowledge (36). Studies emphasize that factors such as trust in institutions, exposure to misinformation, and the quality of medical education play a crucial role in shaping vaccination attitudes (37). Moreover, vaccine hesitancy among students is influenced by socio-demographic and contextual variables, including social media exposure, highlighting the need for targeted educational interventions (38).

A study by Ulbrichtova et al. (39) conducted among healthcare and non-medical personnel in northern Slovakia showed that the level of vaccination acceptance was high, but a significant proportion of respondents justified their refusal to vaccinate with fear of side effects and low trust in information campaigns. In turn, Tatarkova et al. (40), in a study comparing the attitudes of teachers and outpatient physicians in Slovakia, showed that high willingness to vaccinate (85.5%) was motivated primarily by professional responsibility and the desire to protect the family. Sovicova et al. (41), in a study conducted among Slovak medical students, found that as many as 96.1% had a positive attitude toward vaccination against COVID-19, and the main factor favoring vaccination acceptance was a sense of professional obligation and trust in science. Similar findings were reported in our study, where the main reason for vaccination acceptance was the belief in its effectiveness, both among Slovak and Polish students (Slovakia: 105; 61.4%, Poland: 186; 57.23%).

In our own study, the most common negative impact of the pandemic for Polish and Slovak students was the lack of parties/social gatherings (Slovakia: 128; 62.75%, Poland: 282; 65.43%). However, responses such as being confinement at home, family arguments, and restaurant closures were also common. Considering the most burdensome restrictions during the pandemic, the ban on parties and social gatherings proved to be the most burdensome for all surveyed students. The same applies to the lack of places and activities—students in these groups also most frequently indicated that the lack of parties and social gatherings was felt most acutely.

A review by Fadlallah et al. (42) identified numerous unintended side effects of restrictions, including deterioration of mental health, economic disruption, and limited access to medical services. Nguyen et al. (43) described deterioration of physical activity, diet, and overall lifestyle as a result of restrictions, particularly among students and young adults. The results of the study by Cascini et al. (44) showed that global acceptance of restrictions was strongly dependent on the level of trust in state institutions and the communication competence of the authorities.

Although the COVID-19 pandemic has had numerous negative health, mental, and social impacts, the literature also indicates its indirect positive effects. In our study, Slovak students most often responded that during the pandemic they had more time to develop their interests (106; 51.96%) and family life (120; 58.82%). Polish students, on the other hand, most often had more time to read books (182; 42.33%). Slovak students, like Polish students, spent most of their time during the COVID-19 pandemic on self-care (Slovakia: 148; 72.55%, Poland: 242; 56.15%) and self-development (Slovakia: 118; 57.84%, Poland: 243; 56.38%).

In a study by Przymuszała et al. (45) on remote medical education, students positively assessed the flexibility and accessibility of online learning, but noted deficits in practical clinical skills. Gebeyehu et al. (46) in their study emphasized that in many countries the pandemic accelerated the digital transformation in healthcare, improved the organization of services, and increased awareness of the importance of prevention. Some analyses also noted a short-term improvement in environmental quality (e.g., a reduction in NO₂ and particulate matter concentrations).

All observed above-mentioned differences between students from Poland and Slovakia should not be interpreted solely as objective assessments of healthcare systems, but rather as subjective perceptions shaped by broader contextual factors. These perceptions may be influenced by differences in the organization and functioning of healthcare systems, as well as by country-specific approaches to managing the COVID-19 pandemic. Variations in access to healthcare services, waiting times, and the structure of care delivery may have contributed to differing evaluations among students.

In addition, public health communication strategies and the clarity and consistency of pandemic-related messaging may have played an important role in shaping students’ attitudes and perceptions. Differences in the way information was communicated by authorities, as well as exposure to misinformation or conflicting messages, may have influenced both trust in the healthcare system and attitudes toward vaccination.

The level of trust in public institutions is another important factor that may explain the observed differences. Previous studies have shown that trust in government and healthcare authorities is strongly associated with compliance with public health measures and acceptance of vaccination, and may also influence how individuals evaluate the effectiveness of healthcare systems during crisis situations.

Furthermore, educational and experiential factors should be taken into account. Medical and nursing students differ in their level of clinical exposure, knowledge, and engagement with healthcare systems, which may shape their perceptions of both healthcare quality and pandemic management. These differences may be further modified by national educational frameworks and the extent to which students were involved in clinical or pandemic-related activities.

Taken together, these findings suggest that the differences observed between Poland and Slovakia likely reflect a complex interplay of systemic, cultural, and educational factors rather than purely objective differences in healthcare quality. Therefore, the results should be interpreted with caution and understood within the broader socio-contextual framework.

However, an important aspect of the present study is the timing of data collection. The data were collected in the academic year 2024-2025, i.e., in the post-pandemic period. The study included students who were enrolled at universities at the time of the survey but did not study during the most critical phases of the COVID-19 pandemic. Consequently, their perceptions are based on retrospective experiences, shaped not only by direct personal memories but also by subsequent information, social narratives, and later academic exposure. This temporal distance may influence the way the pandemic is evaluated, potentially introducing recall bias and reinterpretation of past events. Therefore, the findings should be interpreted with caution, as they reflect *post hoc* perceptions rather than real-time experiences of students functioning within academic and healthcare systems during the pandemic.

Although several demographic and educational variables were collected, including sex, field of study, place of residence, and year of study, they were not included in multivariate analyses. These factors may have influenced the observed results and should therefore be considered as potential confounders.

Previous research indicates that sex differences may affect health-related attitudes and behaviors, including risk perception, compliance with preventive measures, and vaccination uptake. Similarly, field of study and level of education may be associated with differences in knowledge, clinical exposure, and trust in healthcare interventions, which in turn can influence perceptions of the healthcare system and attitudes toward vaccination.

In addition, place of residence may play a role in shaping access to healthcare services, exposure to health information, and trust in public institutions. Students from urban and rural areas may therefore differ in their evaluation of healthcare quality and public health measures.

The year of study may also be relevant, as more advanced students are more likely to have direct clinical experience, which can shape their understanding of healthcare systems and public health responses.

The lack of control for these variables limits the ability to isolate the effects of country-level differences and may lead to an oversimplified interpretation of the results. Therefore, future studies should consider incorporating multivariate analyses to better account for the complex interplay of individual and contextual factors.

### 4.1 Strengths and limitations of the study

In the manuscript, we have included the strengths of the study and its limitations.

The study’s main strengths include the collection of extensive empirical data on the perception of the COVID-19 pandemic among medical students. The comparative nature of the analysis, encompassing two culturally similar populations, is also significant, enabling the identification of differences in the systemic context.

The study considers various dimensions of pandemic perception, including assessments of healthcare quality and attitudes toward vaccination, allowing for a more comprehensive understanding of the phenomenon under analysis.

The study has also several limitations that should be considered when interpreting the results. First, convenience sampling based on self-selection was used, which limits the representativeness and generalizability of the results and may be associated with selection bias.

Second, the data are based on self-report, which carries the risk of being influenced by social desirability and social pressure, particularly regarding attitudes toward vaccination.

Furthermore, due to the retrospective nature of the study, there is a possibility of recall bias. The heterogeneity of the study group in terms of when participants began their studies may have further influenced the responses.

Finally, demographic and educational data were not included in multivariate analyses, meaning that potential confounding variables such as gender, field of study, or stage of education were not controlled for. No stratified analyses according to demographic or educational variables were performed, which may limit the interpretation of subgroup-specific differences.

Additionally, the original tool used has not been fully psychometrically validated, which may limit the reliability and validity of the measurement.

## 5 Conclusion

The findings provide exploratory insight into retrospective perceptions related to psychosocial experiences during the COVID-19 pandemic among surveyed medical students from Poland and Slovakia. The lower evaluation of healthcare services during the pandemic and differences observed between Poland and Slovakia may reflect the perceived strain on healthcare systems, although these results should be interpreted with caution.

While students expressed confidence in vaccination, differences between declared attitudes and vaccination uptake were observed, which may highlight the possible role of targeted educational and communication strategies. Overall, the findings may suggest the importance of considering psychosocial and systemic factors in shaping health-related attitudes among medical students. However, due to study limitations, the results should be interpreted cautiously and are not fully generalizable and should primarily be understood within the context of the analyzed sample.

## Data Availability

The raw data supporting the conclusions of this article will be made available by the authors, without undue reservation.
